# Diabetes education improves depressive state in newly diagnosed patients with type 2 diabetes

**DOI:** 10.12669/pjms.295.3573

**Published:** 2013

**Authors:** Bin Chen, Xiyao Zhang, Xiuping Xu, Xiaofeng Lv, Lu Yao, Xu Huang, Xueying Guo, Baozhu Liu, Qiang Li, Can Cui

**Affiliations:** 1 Bin Chen, Department of Endocrinology and Metabolism, The Military General Hospital of Beijing PLA, Beijing 100700, China.; 2 Xiyao Zhang, Department of Endocrinology and Metabolism, The 2nd Hospital affiliated to Harbin Medical University, No. 246 of Nangang, District, Harbin 150080, China.; 3 Xiuping Xu, Department of Endocrinology and Metabolism, The Military General Hospital of Beijing PLA, Beijing 100700, China.; 4 Xiaofeng Lv, Department of Endocrinology and Metabolism, The Military General Hospital of Beijing PLA, Beijing 100700, China.; 5 Lu Yao, Department of Endocrinology and Metabolism, The Military General Hospital of Beijing PLA, Beijing 100700, China.; 6 Xu Huang, Department of Endocrinology and Metabolism, The 2nd Hospital affiliated to Harbin Medical University, No. 246 of Nangang, District, Harbin 150080, China.; 7 Xueying Guo, Department of Endocrinology and Metabolism, The 2nd Hospital affiliated to Harbin Medical University, No. 246 of Nangang, District, Harbin 150080, China.; 8 Baozhu Liu, Department of Endocrinology and Metabolism, The 2nd Hospital affiliated to Harbin Medical University, No. 246 of Nangang, District, Harbin 150080, China.; 9 Qiang Li, Department of Endocrinology and Metabolism, The 2nd Hospital affiliated to Harbin Medical University, No. 246 of Nangang, District, Harbin 150080, China.; 10 Can Cui, Department of Endocrinology and Metabolism, The 2nd Hospital affiliated to Harbin Medical University, No. 246 of Nangang, District, Harbin 150080, China.

**Keywords:** Incidence of depression, emotional distress, Type 2 diabetes, diabetes education

## Abstract

***Objectives:*** The prevalence of depression is relatively high in individuals with diabetes. However, screening and monitoring of depressive state in patients with diabetes is still neglected in developing countries and the treatment of diabetes-related depression is rarely performed in these countries. In this study, our aim was to study the role of diabetes education in the improvement of depressive state in newly diagnosed patients with type 2 diabetes.

***Methods:*** The Dutch version of the center for epidemiological studies depression scale (CES-D scale) and the problem areas in diabetes (PAID) questionnaire were used to assess depression and diabetes-specific emotional distress in 1200 newly diagnosed male adult patients with type 2 diabetes before and after a two-week diabetes education by professionally trained nurses. Pearson correlation and regression analysis were used to analyze the factors related to depression in patients with type 2 diabetes.

***Results:*** The incidence of depression in newly diagnosed patients with type 2 diabetes was 28%, and the rate of diabetes-specific emotional distress was 65.5%. High education levels, low income were correlated to depression in individuals with diabetes. After two weeks of diabetes education, the incidence of depression and diabetes-specific emotional distress decreased significantly to 20.5% (P < 0.05) and 11% (P < 0.001), respectively.

***Conclusions:*** The incidence of depression, especially diabetes-specific emotional distress, was relatively high in newly diagnosed patients with type 2 diabetes. The depression state could be improved by diabetes education.

## INTRODUCTION

Depression is not only associated with impaired life quality,^1^ but also with poor glycaemic control, increased risks for the development of diabetes complications and higher mortality rates.^2^^-^^4^ Although the relationship between depression and diabetes is not fully understood and many studies are still going on, the high healthcare expenditure^5^ and increased mortality^6^^,^^7^ caused by depression indicated that all patients with diabetes should undergo regular screening for depression.

The prevalence of depression is relatively high in patients with type 1 and type 2 diabetes.^8^^,^^9^ Compared with non-diabetic individuals, patients with type 2 diabetes have a 24% increased risk of developing depression.^10^ The reasons for the increased prevalence and incidence of depression in diabetes patients are still poorly understood, and it remains unclear whether the presence of patients with diabetes increases risk for depression, or whether depression increases risk for diabetes.^11^

Most of depression in patients with type 2 diabetes has not been screened and the treatment of depression in patients with diabetes is in an even poorer state. Though there are some studies that investigated the occurrence of depression nowadays^12^^-^^15^, none of them assessed the duration of depressive episodes. Therefore, these studies didn’t provide insight into the dynamic changes of depression in diabetes.^10^

In this study, we investigated the incidence of depression in patients who were newly diagnosed with type 2 diabetes and the changes on depressive state after a two-week diabetes education in northeast China.

## METHODS

***Subjects:*** In this study, 1200 male adults with newly diagnosed type 2 diabetes from northeast China were included between Aug. 2008 and Nov. 2012. This study was conducted in the 2^nd^ Hospital affiliated to Harbin Medical University, Harbin, China and the Military General Hospital of Beijing PLA, Beijing, China. None of the patients had hyperglycemia and none of them had been assessed or diagnosed as depression before. Ethical approval for the research was obtained from the Medical Ethical Committee of the Harbin Medical University, and informed consents according to the Declaration of Helsinki were obtained in all cases. Patients with acute complications of diabetes mellitus, such as diabetic ketoacidosis, heperglycemic hyperosmolar state and serious hypoglycemia, were excluded while those with microvascular complications of diabetes were also not enrolled. Patients with cardiovascular and cerebra-vascular disease or symptomatic peripheral arteries disease were also not included. The exclusion criteria also included the patients with other system chronic somatic diseases. The demographic and clinical characteristics, such as age, education background, socioeconomic status, marital status and recent HbA1c were shown in Table-I:

**Table-I:** Demographic characteristics of patients

	*Number*	*Percent(%)*	*Average * *(means* *±* *SD)*
***Age strata*** *** (yrs)***			
20-40yrs	345	28.8	50.5±14.2
41-60yrs	697	58.1	
>60ysr	158	13.1	
***Education background***			
Illiterate	45	3.80	
Up to 9 years of school	484	40.3	
>9year of school	671	55.9	
Over high school	484	40.3	
Over university	296	24.7	
***Socioeconomic status month salary ($)***			
<300	317	26.4	311.7 ± 140.6
301-800	634	52.8	
>800	249	20.8	
***Marital status***			
Living with spouse	1067	88.9	
Not living with spouse	133	11.1	
***HbA1c (%)***			
7-9	256	21.3	8.4±2.8
9-11	578	48.2	
>11	366	30.5	

Note: yrs, years; $, USD

***Assessment of Depression:*** To assess symptoms of depression, the Dutch version of the center for epidemiological studies depression scale (CES-D scale) was used.^16^^,^^17^ This is a 20-item, self-report scale that asks respondents to indicate the frequency of occurrence of 20 depressive symptoms during the previous week. The instrument uses a four-point response set, ranging from ‘rarely or none of the time’ to ‘most of the time or always’. Higher scores indicate more depressive symptoms and a cut-off point of 16 or more is generally accepted as indicative of a clinically significant level of depression symptoms.^16^^,^^17^ The automated World Health Organization Composite International Diagnostic Interview (CIDI-auto) is a structured diagnostic interview, which was used to determine whether the patients suffered from a depressive disorder and/or an anxiety disorder, according to Diagnostic and Statistical Manual-V (DSM-V), DSM-IV criteria.^18^^-^^20^ In our study, lay interviewers, medical graduate students and registered nurses, were trained by a certified psycho-professional consultant to use the CIDI-auto.

***Assessment of diabetes-specific emotional distress:*** Diabetes specific emotional distress was assessed using the Dutch version of the PAID survey,^21^^,^^22^ which was inquired by the interviewer. This questionnaire consists of 20 items, which can be rated on a five-point scale ranging from 0 (no problem) to 4 (serious problem). Examples of items are: ‘Not accepting diabetes’, ‘Worrying about the future and the possibility of serious complications’, ‘Feeling overwhelmed by your diabetes regimen’ and ‘Feeling alone with diabetes’. Higher scores indicate more serious emotional problems.

***Standard diabetes education (SDE):*** The first assessment of depression and diabetes-specific emotional distress were administrated to the patients within their first two days in hospital by the method described above. After two-week management of glyceamic control, including diabetes education for the patient by professional nurses, the second assessment was administrated. Diabetes education includes three aspects. First, there were classes of diabetes in which the patients comprehended the general concepts of diabetes, including definition, classification, symptoms, diagnostic standard, complications and management of diabetes. Second, there was nurses talking with each patient individually to explain questions for the patient daily and making an individualized diabetic care program for each patient. Third, there were workshops for all patients every Thursday in order to encourage the patients to communicate with each other to share their experiences.

To exclude the influence of glyceamia control on depressive state, 40 patients with depression were selected and divided into two groups randomly after first assessment of depression. One group took part in the diabetes education routinely, while the other group did not followed the program of diabetes education. The groups were paired with demographic and clinical characteristics. Two weeks later, all the 40 patients were assessed on depression state for the second time.

***Statistical analysis:*** All the data were analyzed by using SPSS 16.0 package (Chicago, IL, USA) and shown as mean ± SD. Pearson correlation and regression analysis were used to analyze the factors related to depression in diabetes. Students’t test was applied to compare the changes of depression status in patients with diabetes before and after two weeks of diabetes education.

## RESULTS

***Depression incidence in the newly diagnosed patients with type 2 diabetes:*** During the first assessment, 326 patients of the 1200 newly diagnosed patients with type 2 diabetes were considered as depressed according to the questionnaires and the incidence of depression was 28% (Table-II). Moreover, more patients (65.5%) suffered from diabetes-specific emotional distress (Table-II).

**Table-II:** Incidence of depression and diabetes-specific emotional distress in patients with type 2 diabetes

	*before SDE*		*after SDE*	
	*Number*	*Percent*	*Number *	*percent*
*Incidence of depression*				
Total	326	28.0	246*	20.5
mild	109	9.10	129	10.8
moderate	159	13.3	79**	6.58
severe	58	4.80	38*	4.20
Incidence of diabetes-specific emotional distress (%)	786	65.5	132**	11

Note: *, P < 0.05, **, P < 0.001 compared with the incidences before SDE. No., number; SDE, standard diabetes education.

***Related factors to depression in newly diagnosed type 2 diabetes:*** To investigate the factors related to depression in patients with diabetes, pearson correlation and regression analysis were used. To exclude the influence of gender, female patients were not included in the investigation. Results showed that age and marital status were not associated with depression, while education background, socialeconomic status and hyperglyciemia were related to depression. The incidence of depression was higher in those with education background of above university (r=0.405, P < 0.001). Next factors related to diabetes-specific emotional distress were studies. PAID questionnaire scores were 78±26 in those with higher education levels (above university), while the scores were relative lower in the patients with lower education levels (under high school), which is 45 ± 19 (P = 0.012).

Income levels was negatively related to depression in newly diagnosed patients with type 2 diabete (r = 0.303, P = 0.000), while there was no correlation of income levels to diabetes-specific emotional distress (r = 0.8303, P = 0.120). HbA1c levels were positively associated with both depression (r = 0.475, P = 0.002) and diabetes-specific emotional distress (r = 0.512, P = 0.0012).

***Dynamic changes of depression in newly diagnosed patients with type 2 diabetes after two weeks of diabetes education: ***To further assess the effect of diabetes education on diabetes related depression, a two-week diabetes education of all patients was performed as described above. Both the status of depression and diabetes-specific emotional distress were improved significantly in the newly diagnosed patients with type 2 diabetes after the education. The number of patient with depression decreased from 326 to 248, and for diabetes-specific emotional distress the decrease was more significant (Table-II) (P=0.002 and 0.000, respectively). The numbers of patients with moderate or severe depression after SDE were decreased when compared with the numbers before SDE (159 versus 79, 58 versus 38), while the number of patients with mild depression was increased (109 versus 129) (Table-II). 

To gain insight into the influence of glycemia control, 40 patients with depression were selected and divided into two groups with one half taking part in diabetes education while the other didn’t. The scores for CES-D scale in the subjects who did not follow the program of diabetes education were much higher than those taking part in diabetes education program for two weeks (P<0.001) (Table-III). There were no significant differences in HbA1c levels between the two groups both before and after two weeks of education (Table-III).

**Table-III:** Role of diabetes education on depressive state in type 2 diabetes

	*With SDE*	*Without SDE*
Total No.	20	20
Age	40.5±12.3	42.3±9.8
Marital status	Living with spouse	Living with spouse
Education background	Over university	Over university
Monthly salary($)	300-800	300-800
CES-D scale		
Before SDE	41±16	39±12
After SDE	18±16**	34±13^##^
HbA1c		
Before SDE	8.1±0.8	8.2±1.2
After SDE	7.8±1.5	8.0±0.9

Note: **, P < 0.001 compared with the depression score before SDE. ^##^, P < 0.001 compared with the depression score of groups with depression with SDE. No., number; SDE, standard diabetes education

## DISCUSSION

In this study, we showed that the incidence of depression in newly diagnosed male patients with type 2 diabetes was 28 percent, and the diagnostic rate of diabetes-specific emotional disorders in these patients was 65 percent. However, the depression rate in patients with diabetes apparently increased compared with the data of previous reports.^8^^-^^10^ The possible explanation might be that both people with diabetes and healthcare professionals have become increasingly aware of symptoms of depression in diabetes. In addition, measurement of depression varied among studies. For example, different diagnostic criteria and different cut-off values were used for self-report questionnaires. Difference in population involved is also a reason for heterogeneity of the results. In a recent report from India, the prevalence of depression was 49% (95%CI 39.1-58.9)^23^, in which the percentage was higher than our results. Moreover, this is the first time to take depression assessment in such a large scale of patients in China, since the assessment is still not a regular screening process in patient with diabetes in China. It is speculated that if depression assessment were taken as a regular screening in patients with diabetes the incidence of depression related to diabetes, especially the diabetes-specific emotional distress, may be higher than the estimated now, since in that case physicians are possibly to be more sensitive to evaluate depression. 

We found that the education levels and the risk of suffering from depression had positive correlation, which was contrary to the general concept.^24^ During the process of diabetic care program including diabetes education, the nurses for patients with different education background had similar professional knowledge. The patients with higher education levels (above high school) could understand the scale contents quickly and easily, but their compliance was not good for they would like to compare the methods and make some trials, which might be the reason for their sensitivity to depression. However, the trend needs further evidence to exclude that whether it was only a bias. 

It is not strange that economic status is negatively correlated with depressive status. A study in Australia showed that moderate-severe depression was positively associated with socioeconomic disadvantage in diabetes population.^24^ Since the national medical assurance has not protected all people in China, economic status plays an important role in diabetic control. Patient with lower income might suffer from more distress than those with high income. Low income might also be one reason for insufficient diabetic control and other chronic life style diseases, because life-long management is demanded once the diseases diagnosed.

Poorly controlled diabetes is also a high risk factor of depression in patients with diabetes. We found that higher HbA1c levels correlated with higher incidence of depression, which was similar to other scientist’s result.^23^ Although all the patients were newly diagnosed, it is still unclear whether high levels of plasma glucose is the cause or result of depression. A new report showed that anti-depression treatment, such as clomiprimpine, was associated with the prevalence of diabetes in depression population.^25^ The relationship between diabetes and depression was not a simple cause-result issue.^26^ Therefore, long term dynamic studies on depressive episodes are needed.

High education background, low income state and high plasma glucose levels were correlated to depression in newly diagnosed patients with type 2 diabetes. Diabetes education is an important aspect in diabetic care. Although patients could get information through internet, professional diabetes classes are still irreplaceable^23^. Our data showed that after two weeks of diabetes education, the depression rate in the patients was decreased significantly. Nearly 30 percent of patients with depression recovered. Diabetes-specific emotional disorders were improved more significantly after the education, which was decreased from 65 percent to 10 percent after diabetes education. During the process, none of the patients had been treated with anti-depressive medicine and no psyco-interfere measurements were taken. It is suggested that a large portion of depressive state, especially diabetes-specific emotional disorders, had resulted from the misunderstanding of diabetes. Although education on diabetic patients is a key part of the management of diabetes, education and professional consultants are deficient in developing counties. The numbers of patients with moderate or severe depression after SDE were decreased when compared with the numbers before SDE, while the number of patients with mild depression was increased. The reason was that some patients with moderate or severe depression before SDE were improved to mild depression after SDE. 

Since the data showed that glycemia control was negatively correlated to depression in diabetes^23^, the improvement of depressive state in the subjects might be a result of glycemia control, but not the diabetes education. To exclude such possibility, 40 patients with depression were selected and divided into two groups randomly to follow diabetes education program or not. The results proved that diabetes education was related to the improvement of depression in newly diagnosed patients with type 2 diabetes. In conclusion, diabetes education could improve the state of depression, especially diabetes-specific emotional distress, in patients with diabetes.

In this study, a time-limited (two-week) diabetes education was provided by professionally trained nurses. In the future, long-term diabetes education such as four-week and six-week education will be provided. Studies of possible changes in levels of key hormones or cellular factors related to depression and emotional distress in patients will also be performed in the future. 

## CONCLUSIONS

The incidence of depression, especially diabetes-specific emotional distress, was relatively high in newly diagnosed patients with type 2 diabetes. The education background, social-economic state and plasma glucose levels were factors correlated to depression in newly diagnosed patients with type 2 diabetes. The depression state could be improved by diabetes education.
